# Different roles of protein biomarkers predicting eGFR trajectories in people with chronic kidney disease and diabetes mellitus: a nationwide retrospective cohort study

**DOI:** 10.1186/s12933-023-01808-5

**Published:** 2023-03-29

**Authors:** Michael Kammer, Andreas Heinzel, Karin Hu, Heike Meiselbach, Mariella Gregorich, Martin Busch, Kevin L. Duffin, Maria F. Gomez, Kai-Uwe Eckardt, Rainer Oberbauer, for the BEAt-DKD consortium

**Affiliations:** 1 Department of Internal Medicine III, Division of Nephrology and Dialysis, Medical University of Vienna, Währinger Gürtel 18-20, 1090 Vienna, Austria; 2 Center for Medical Data Science, Institute of Clinical Biometrics, Medical University of Vienna, Vienna, Austria; 3 Department of Nephrology and Hypertension, Friedrich-Alexander Universität Erlangen-Nürnberg, Erlangen, Germany; 4 Department of Internal Medicine III, University Hospital Jena, Friedrich-Schiller Universität, Jena, Germany; 5 Lilly Research Laboratories, Eli Lilly and Company, Indianapolis, IN USA; 6 Department of Clinical Sciences, Lund University Diabetes Centre, Lund University, Malmö, Sweden; 7 Department of Nephrology and Medical Intensive Care, Charité Universitätsmedizin Berlin, Berlin, Germany

**Keywords:** Chronic kidney disease, Diabetes mellitus, Prognosis, Proteomics

## Abstract

**Background:**

Chronic kidney disease (CKD) is a common comorbidity in people with diabetes mellitus, and a key risk factor for further life-threatening conditions such as cardiovascular disease. The early prediction of progression of CKD therefore is an important clinical goal, but remains difficult due to the multifaceted nature of the condition. We validated a set of established protein biomarkers for the prediction of trajectories of estimated glomerular filtration rate (eGFR) in people with moderately advanced chronic kidney disease and diabetes mellitus. Our aim was to discern which biomarkers associate with baseline eGFR or are important for the prediction of the future eGFR trajectory.

**Methods:**

We used Bayesian linear mixed models with weakly informative and shrinkage priors for clinical predictors (n = 12) and protein biomarkers (n = 19) to model eGFR trajectories in a retrospective cohort study of people with diabetes mellitus (n = 838) from the nationwide German Chronic Kidney Disease study. We used baseline eGFR to update the models’ predictions, thereby assessing the importance of the predictors and improving predictive accuracy computed using repeated cross-validation.

**Results:**

The model combining clinical and protein predictors had higher predictive performance than a clinical only model, with an $R^{2}$ of 0.44 (95% credible interval 0.37–0.50) before, and 0.59 (95% credible interval 0.51–0.65) after updating by baseline eGFR, respectively. Only few predictors were sufficient to obtain comparable performance to the main model, with markers such as Tumor Necrosis Factor Receptor 1 and Receptor for Advanced Glycation Endproducts being associated with baseline eGFR, while Kidney Injury Molecule 1 and urine albumin-creatinine-ratio were predictive for future eGFR decline.

**Conclusions:**

Protein biomarkers only modestly improve predictive accuracy compared to clinical predictors alone. The different protein markers serve different roles for the prediction of longitudinal eGFR trajectories potentially reflecting their role in the disease pathway.

**Supplementary Information:**

The online version contains supplementary material available at 10.1186/s12933-023-01808-5.

## Background

The prevalence of metabolic syndrome and diabetes mellitus (DM) is on the rise worldwide in adults, adolescents and even in children [1–4]. Chronic kidney disease (CKD) is a common comorbidity in these people, and a key risk factor for life limiting conditions such as arterial hypertension and cardiovascular disease. In the last decade effective treatments emerged that reduce the risk of progression for CKD [5–7], making an accurate, early prediction of the highly variable individual decline of kidney function in terms of estimated glomerular filtration rate (eGFR) an important clinical goal. The combination of clinical predictors with plasma biomarkers was found to improve predictive accuracy for individual eGFR loss in early stages of the disease, but so far showed limited clinical utility [8–19]. Furthermore, few studies addressed how the biomarkers contributed to the predictions. Kidney Injury Molecule 1 (KIM1) for example has been shown in experimental models of kidney injury and human studies to be an intrinsic kidney injury marker whereas other markers such as Tumor Necrosis Factor Receptor 1 (TNFR1) represent filtration markers even in settings without intrinsic kidney damage [20, 21]. Therefore, it is likely that different biomarkers contribute differently to the prediction of longitudinal eGFR trajectories, i.e. some may be strongly associated with values close to the baseline, while others may be predictive for future eGFR decline.

To better understand the roles of established plasma biomarkers the specific aims of our study were to validate and discern predictors associated with baseline eGFR and future eGFR decline, as well as to assess their predictive abilities in combination with clinical predictors. We made use of Bayesian linear mixed models to analyze data from persons with diabetes in the German Chronic Kidney Disease (GCKD) study, one of the largest prospective cohort studies of people with moderately advanced CKD [22].

## Methods

### Study design and outcome of interest

We determined eGFR according to the CKD-EPI creatinine equation [23]. To validate the set of selected protein biomarkers, our first objective was to use baseline values of biomarkers and clinical predictors to prognosticate the entire longitudinal eGFR trajectory, thereby assessing the predictive capabilities of these data independently of baseline eGFR. To discern the roles of the predictors, our second objective was to elucidate the added long-term predictive capabilities of the biomarkers on top of baseline eGFR, which is most relevant to clinical practice.

### Study cohort

We analyzed the subcohort of people with DM in the GCKD study, a prospective observational nationwide cohort study in Germany of people under regular nephrological care without the need for kidney replacement therapy [22]. The study did a long-term observation with yearly visits, alternating between in person visits and telephone interviews until year six. It is one of the world’s largest long-term observational CKD cohort studies with more than 5000 patients enrolled between March 2010 and March 2012.

**Fig. 1:**
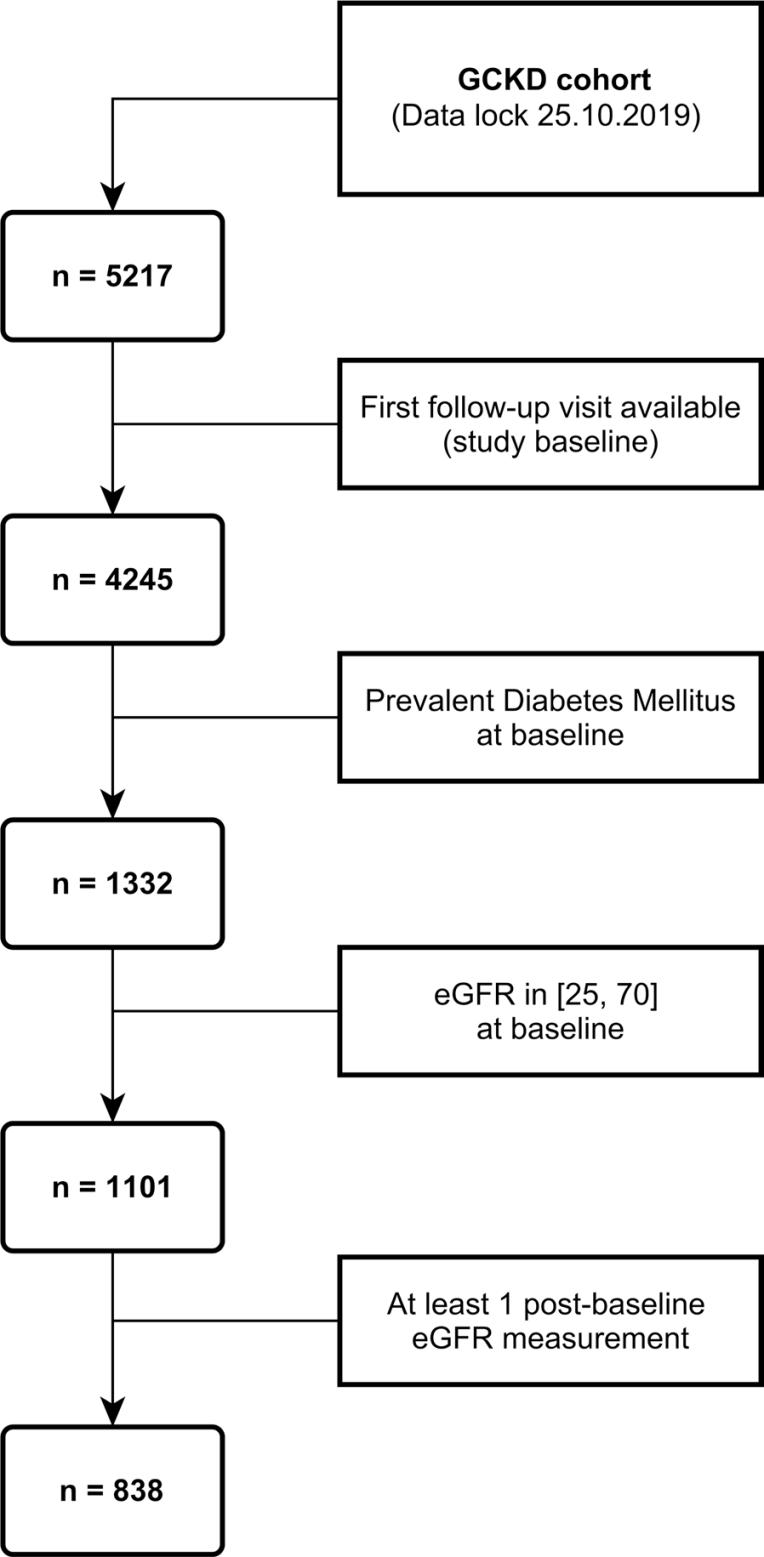
Flowchart of participant inclusion

The inclusion criteria for the GCKD study were an eGFR of 30–60 ml/min/1.73m^2^ or an eGFR > 60 ml/min/1.73m^2^ with overt albuminuria (defined as albumin excretion > 300 mg/g creatinine, protein excretion > 500 mg/g creatinine, or corresponding values for 24 h urinary excretion). Exclusion criteria comprised non-Caucasian ethnicity, solid organ or bone marrow transplantation, active malignancy within 24 months prior to screening, heart failure of New York Heart Association Stage IV, and inability to provide consent. Due to limitation on sample availability, the first in-person follow-up visit two years after enrolment into the GCKD study was referred to as “baseline”, and defined time zero for all computations of observation times in the remainder of this manuscript. Additional inclusion criteria for our study cohort on top of those for the GCKD study were diagnosis of DM, an eGFR of 25–70 ml/min/1.73m^2^ to reflect the natural decline of eGFR between enrolment into GCKD and our analysis baseline, and at least one eGFR measurement post-baseline to contribute to our longitudinal outcome of interest. Persons were defined as diagnosed with DM if they had an HbA1C measurement of at least 6.5%, or if they had a prescription for at least one drug used to treat DM comprising a compound from any class starting with code “A10” (“Drugs used in diabetes”) according to the Anatomical Therapeutic Chemical Classification System [24]. At our baseline, data from 4245 people between 24. January 2012 and 25. October 2019 (data lock) were available, including 1332 with DM. We provide an overview of participant inclusion in Fig. 1.

### Clinical predictors

We analyzed several common clinical predictors: age, sex, body mass index (BMI), smoking status (never / ever), mean arterial pressure (MAP), serum cholesterol, urine albumin-creatinine-ratio (UACR), hemoglobin A1C (HbA_1c_), hemoglobin, intake of blood pressure lowering medication, antidiabetic medication, and lipid lowering medication.

### Protein biomarker selection and measurement

We selected 19 protein biomarkers from a pool of candidates with prior evidence for an association with renal function derived from two recently published studies by Niewczas et al. and Gerstein et al., as well as earlier analyses within the BEAt-DKD and RHAPSODY consortia [10, 12, 13, 15]. We maximized the number of biomarkers that could be measured using a single sample aliquot by optimizing the selection regarding the availability of multiplexed Luminex and ELISA assays.

A Human Premixed Multi-Analyte Luminex Kit (RD-LXSAHM-13, R&D Systems, Minneapolis, USA) was used to measure 13 serum biomarkers with 1:2 sample dilution: Alpha 1-Microglobulin, Angiopoietin-2, C–C motif Chemokine 11 (CCL11), C–C motif Chemokine 15 (CCL15), Chemerin, Fas, Fas Ligand, Growth Differentiation Factor 15 (GDF15), Interleukin 1 Receptor Type 1 (IL1R1), Matrix Metallopeptidase 7 (MMP7), Receptor for Advanced Glycation Endproducts (RAGE), TNFR1, and u-Plasminogen Activator (uPA). An additional Human Premixed Multi-Analyte Luminex Kit (RD-LXSAHM-05, R&D Systems) was used to measure five serum biomarkers with 1:50 sample dilution: Angiopoietin-1, C–C motif Chemokine 5 (CCL5), C–C motif Chemokine 14 (CCL14), Galectin-3 and Myoglobin. Assays were processed following the protocol provided by the manufacturer and measured on a Luminex 200 (Luminex Corporation, Austin, USA) using the xPonent software (Luminex Corporation) with settings recommended in the protocol.

Additionally, KIM1 was measured using an ELISA (RD-DSKM100, R&D Systems). A 1:2 sample dilution was applied and the assay was processed according to the manufacturer’s protocol. Optical density was determined using a TriStar^2^ LB 942 Modular Multimode Microplate Reader (Berthold Technologies, Bad Wildbad, Germany) with the MikroWin2010 software (v5.21, Berthold Technologies) set as instructed in the assay protocol.

All samples were measured as technical replicates. A coefficient of variation (CV) ≤ 15% was required for a measurement to be considered valid. Incurred sample reruns of > 10% of all measured samples were performed on different plates, requiring an inter-plate CV of < 20% to consider the measurements as valid. Three quality control samples (high, medium, low concentration) diluted from the supplied high standard of each assay were included on each measured plate.

Concentrations from raw fluorescence signals outside of the standard range were truncated to fixed values (1/√2 times the lowest or √2 times the highest respective standard value). The measurement with smallest CV was preferred when multiple measurements were available due to reruns.

### Statistical analysis

We report the cohort demographics by medians and interquartile ranges (IQR) for continuous variables, as well as by absolute and relative frequencies for discrete variables.

#### General modeling strategy

We analyzed the longitudinal eGFR trajectories using Bayesian multivariable linear mixed models (BLMM). Such models allow to discern the main term modeling overall eGFR levels (baseline coefficient), and an interaction term with observation time modeling the eGFR decline (slope coefficient) for each independent variable [25]. Person-specific trajectories were modelled using random intercepts and slopes. We fitted several BLMM comprising different variable sets as fixed effects. First, univariable BLMM using single protein biomarkers to assess the univariable association with eGFR. Second, the clinical BLMM using only clinical predictors to serve as a reference model in terms of prediction performance. Third, the main BLMM combining clinical predictors and biomarkers. All models also included observation time and interaction terms with time to model eGFR decline. The univariable and clinical BLMM used weakly informative Student-t distributions as coefficient prior distributions, while the main BLMM used regularized Horseshoe prior distributions to enforce sparsity and shrink the effects of unimportant variables towards zero [26, 27]. All variance parameters used weakly informative priors. We assessed the choice of hyperparameters via sensitivity analyses. Model convergence was evaluated by graphical inspection of the Markov chain traceplots, the $\widehat{R}$ statistic and other sampler diagnostics [28, 29]. We assessed model fit via the normality of residuals and calibration plots.

All biomarker levels and UACR were log2-transformed during modeling to achieve more symmetric distributions. For comparability, coefficients are reported on a standardized scale corresponding to units of standard deviations, and are given as summaries of the model posteriors, i.e. the median of the distribution and a 95% equal tailed Bayesian credible interval (BCI). These intervals represent a contiguous region that contains the unobserved coefficient value with 95% probability, given our modeling assumptions. Model prediction performance via marginal predictions using only fixed effects was assessed in terms of the explained variation $R^{2}$ and the adjusted $R^{2}$ (computed as $1-\frac{(1-R^2)\, (n-1) }{n-p-1}$, with $n$ the number of observations, and $p$ the number of fixed effects), as well as the root mean squared error (RMSE). We used 5-times repeated fivefold cross-validation to estimate the out-of-sample performance.

#### Model update by baseline eGFR

Each model included baseline eGFR as part of the longitudinal outcome (objective 1). However, to reflect the practical use of the models (objective 2) we incorporated baseline eGFR for predictions of future (post-baseline) eGFR for unseen individuals by updating the random coefficient posteriors, i.e. computing the best linear unbiased predictors of the random effects conditional on the observed baseline values [30]. Thereby we prevented over-optimistic model fit when using baseline eGFR as independent variable, but still gained improved prediction performance for the future eGFR trajectory. This also allowed us to elucidate the impact of baseline eGFR on the model’s predictions.

#### Variable importance

We assessed the importance of predictors in the main BLMM for both objectives by ordering them by the increase in cross-validated RMSE when removing a single variable from the full main model and its updated version. Subsequently, we used this ordering to obtain a sequence of nested submodels of the main model, which provide predictions that become incrementally better approximations of the main model predictions as variables are added one-by-one. In detail, we started with a model comprising only the intercept and observation time, and then added more variables (main term and interaction with time) according to the ordering by cross-validated RMSE to obtain incrementally larger models. We computed the submodel predictions using a reference model based projection approach [31–33]. Due to the impact of baseline eGFR the orderings for both objectives differed, discerning the importance of variables as a replacement of baseline eGFR, and for predicting future eGFR in addition to baseline eGFR.

#### Missing data

We used multiple imputation with 20 imputations to account for missing data. All models were fitted in each imputed dataset, and the resulting posteriors pooled to obtain a single posterior incorporating the additional uncertainty due to missing data.

#### Implementation details

We used the R statistical software (version 4.0.4) for all analyses, implementing the BLMM in Stan (version 2.21.0) accessed via the brms package (version 2.16), and the multiple imputation using the mice package (version 3.13) [34]. We provide additional details in the extended Statistical Methods in the Supplementary Material, and considerations regarding sample size in Additional file 1: Figure S1.

## Results

In total, we measured 19 protein biomarkers at baseline in 838 people with DM (predominantly Type 2 DM). Demographics of our study cohort are presented in Table 1. For most participants two post-baseline eGFR measurements were available (n = 525, 63%), and the median observed follow-up after baseline was 3.9 years (IQR [3.5, 4.1]). Overall loss-to-follow-up in the GCKD cohort was low: 45 persons (5%) from our subcohort died during follow-up and 9 (1%) dropped out due to other reasons. The median decline in eGFR, estimated via person-specific regression models, was -0.8 ml/min/1.73m^2^ per year (IQR [− 3.0, 1.1]).

**Table 1:** Cohort demographics of study patients with Diabetes mellitus (n = 838) at study baseline. Data are median and IQR for continuous variables, or absolute and relative frequencies for categorical variables

Variable	Baseline value	Missing
Age (years)	69 [62, 73]	0 (0%)
Sex (female)	287 (34%)	0 (0%)
Body mass index (kg/m^2^)	32 [28, 36]	1 (< 1%)
Smoking status (ever)	505 (50%)	1 (< 1%)
Mean arterial pressure (mmHg)	97 [90, 105]	2 (< 1%)
Serum cholesterol (mg/dL)	192 [164, 225]	0 (0%)
HbA_1c_ (mmol/L)	53 [48, 61]	6 (< 1%)
HbA_1c_ (%)	7.0 [6.6, 7.8]	
Hemoglobin (g/dL)	13.7 [12.6, 14.8]	6 (< 1%)
Serum creatinine (mg/dL)	1.5 [1.3, 1.8]	0 (0%)
Urine albumin-creatinine-ratio (mg/g)	37 [9, 224]	18 (2%)
eGFR (mL/min/1.73m^2^)	42 [35, 51]	0 (0%)
Blood pressure medication (intake)	793 (95%)	0 (0%)
Diabetes medication (intake)	697 (83%)	0 (0%)
Lipid lowering medication (intake)	555 (66%)	0 (0%)

Measured protein biomarker concentrations used in the analysis are depicted in Additional file 1: Figure S2. The proportion of missing biomarker measurements was low at around 3%. We provide an overview of biomarker availability, truncation and measurement issues in Additional file 1: Tables S1 and S2. The Spearman correlation (Additional file 1: Figure S3) between clinical variables (except creatinine and eGFR) and biomarkers was generally low (median 0.03, IQR [− 0.02, 0.07]). In contrast, the correlations between biomarkers and creatinine (0.25 [0.09, 0.35]) or eGFR (− 0.30 [− 0.38, − 0.12]) were higher in magnitude.

### Models for eGFR

All BLMM reported in the following showed satisfactory convergence (Additional file 1: Table S3) and model fit (Additional file 1: Figure S4 shows the main model fit). The results reported here remained unchanged in all our sensitivity analyses (see Extended Statistical Methods in the Supplementary Material).

#### Univariable protein biomarker models

**Fig. 2:**
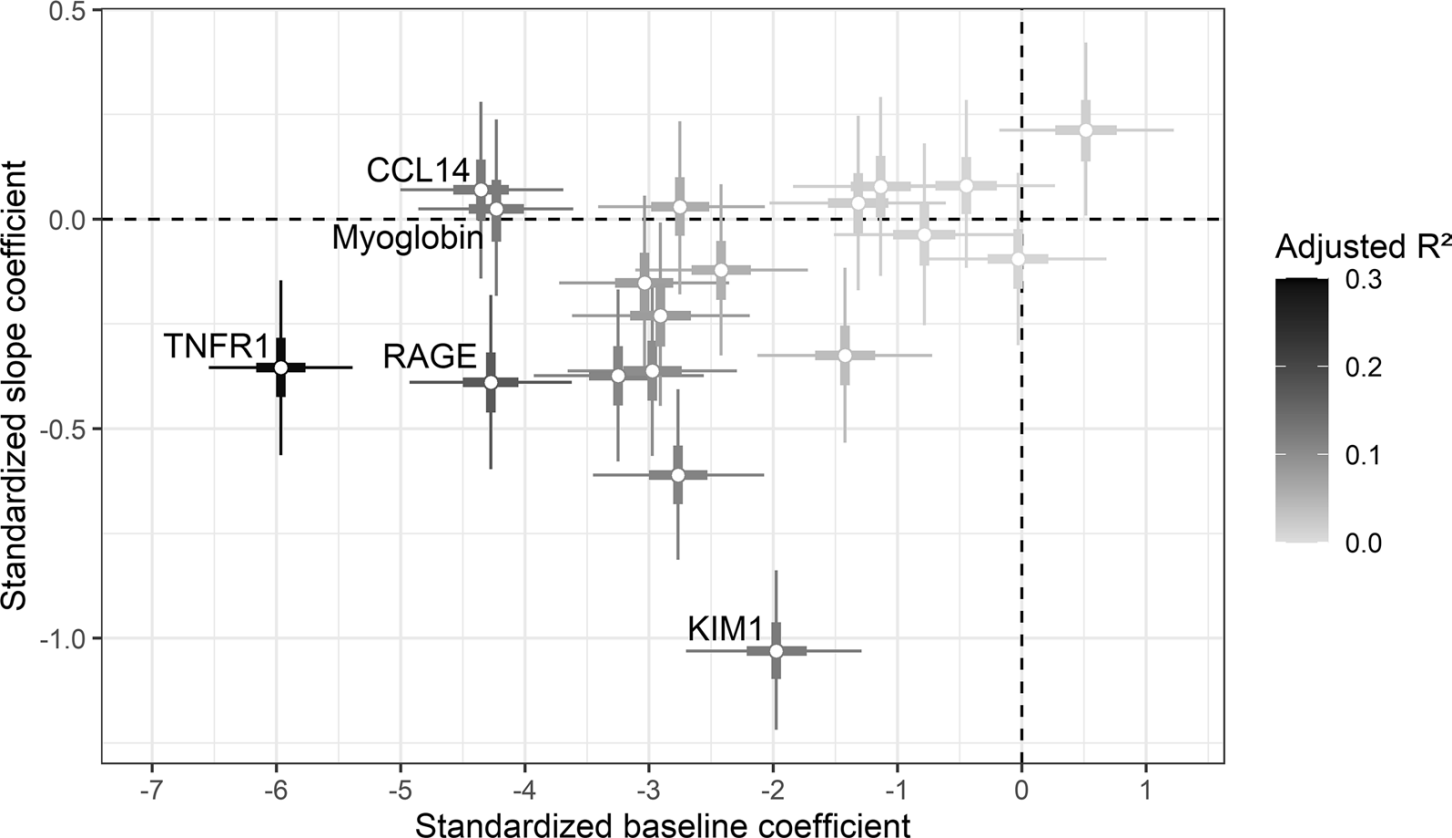
Standardized coefficients estimated by univariable Bayesian linear mixed models. The thin black bars indicate 95% Bayesian credible intervals for the coefficients; the thick black bars indicate 50% Bayesian credible intervals. The intersection point of the horizontal and vertical bars indicated by the point gives the values of the baseline and slope coefficients. The top-5 biomarkers in terms of posterior median adjusted $R^{2}$ pooled over all observation times are annotated in the graphic. Note the different x- and y-axis scales. Most biomarkers are concentrated around the x-axis, indicating an association with baseline eGFR, but weak association with the longitudinal eGFR trajectory

In terms of median posterior adjusted $R^{2}$ pooled across observation time, TNFR1 (0.30, 95% BCI [0.26, 0.33]) and RAGE (0.17 [0.14, 0.21]) showed the strongest associations with eGFR in univariable BLMM. All other markers had adjusted $R^{2}$ values below 0.12, and most of them showed an association via their baseline coefficients (i.e. their 95% BCI excluded zero). For KIM1 (adjusted $R^{2}$ 0.12 [0.09, 0.15]) the standardized slope coefficient had the greatest magnitude of all biomarkers, while for many other markers the association with eGFR trajectory was weak and their 95% BCIs included zero (Fig. 2).

#### Clinical reference model

The model using clinical predictors (12 in total) showed modest predictive performance for the whole eGFR trajectory (objective 1). Its cross-validated median posterior $R^{2}$ was 0.17 (95% BCI [0.11, 0.22], RMSE 11.79 [10.83, 12.66]). Using baseline eGFR to update the model’s predictions (objective 2), the cross-validated performance for post-baseline eGFR values greatly improved with an $R^{2}$ of 0.56 (95% BCI [0.47, 0.62], RMSE 9.00 [8.09, 10.31]). See Additional file 1: Table S4 for a breakdown of performance by follow-up time.

#### Main model

**Fig. 3:**
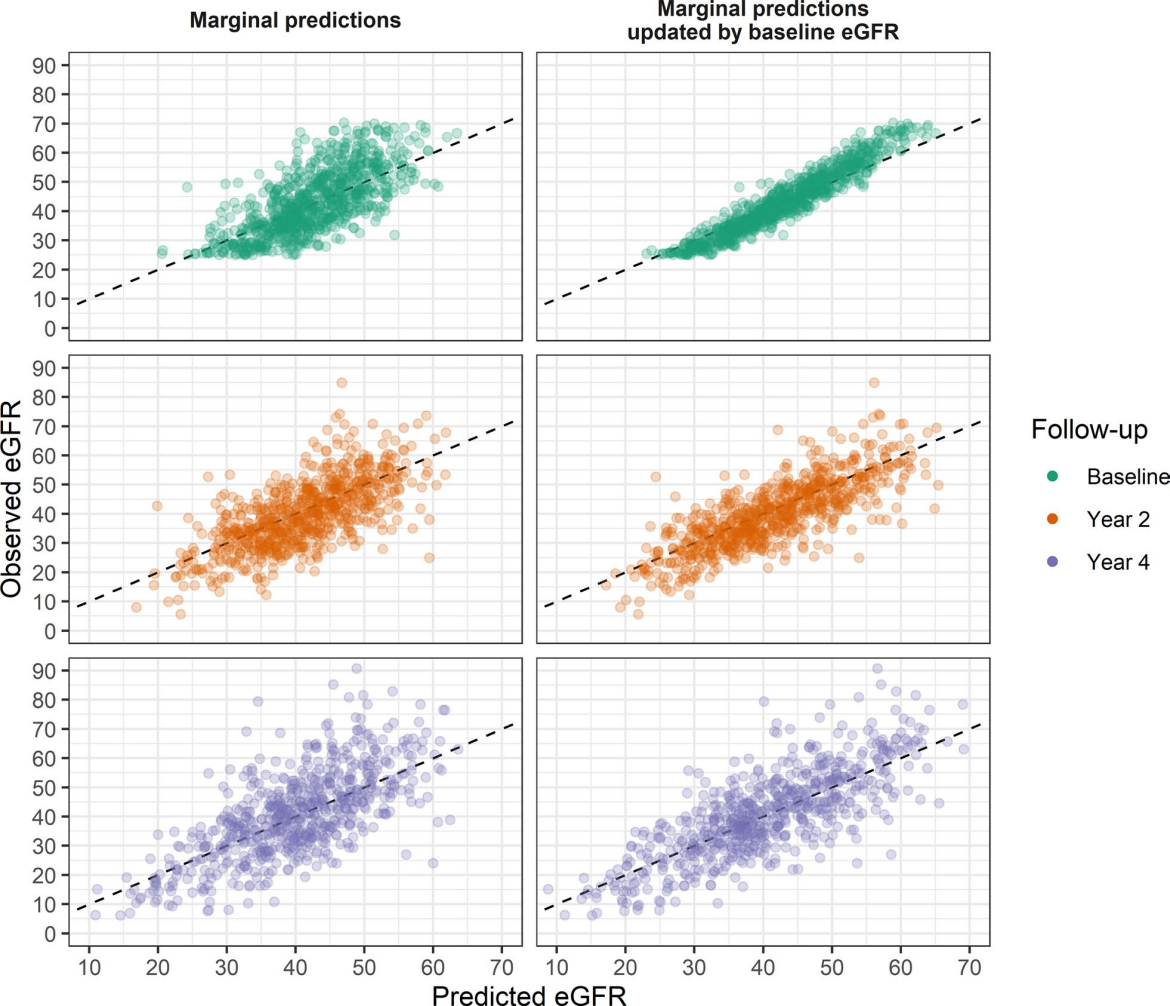
Calibration of posterior median of marginal predictions from the main model, before and after update by baseline eGFR values and stratified by time of observation. Overall, the calibration of predictions was satisfactory over the whole follow-up period. Updating by baseline eGFR led to better calibration and prediction performance, as demonstrated by a more narrow spread around the diagonal line of perfect prediction, even for later follow-up times. The evaluation is stratified by planned follow-up times, actually observed follow-up times used in the model differ slightly. Cross-validated performances by follow-up are reported in Additional file 1: Table S4

The model combining clinical and biomarker predictors (31 in total) had improved predictive performance compared to the clinical model for objective 1. Its predictions were well calibrated, indicating adequate model fit (Fig. 3 and Additional file 1: Figure S4). The cross-validated median posterior $R^{2}$ was 0.44 (95% BCI [0.37, 0.50], RMSE 9.51, 95% BCI [8.60, 10.15]). Predictive performance for post-baseline eGFR was further improved by updating with baseline eGFR for objective 2, with a cross-validated $R^{2}$ of 0.59 (95% BCI [0.51, 0.65], RMSE 8.80, 95% BCI [7.80, 9.95]). See Additional file 1: Table S4 for a breakdown of performance by follow-up time. Many of the predictors’ coefficients were shrunken towards zero (Additional file 1: Table S5 and Additional file 1: Figure S5). In terms of magnitude, TNFR1 had the largest standardized baseline coefficient, followed by other protein biomarkers (RAGE, Myoglobin, CCL14, IL1R1) and age. Only few predictors showed a relevant slope coefficient, with KIM1 and UACR being by far the largest in magnitude.

### Variable importance

**Fig. 4:**
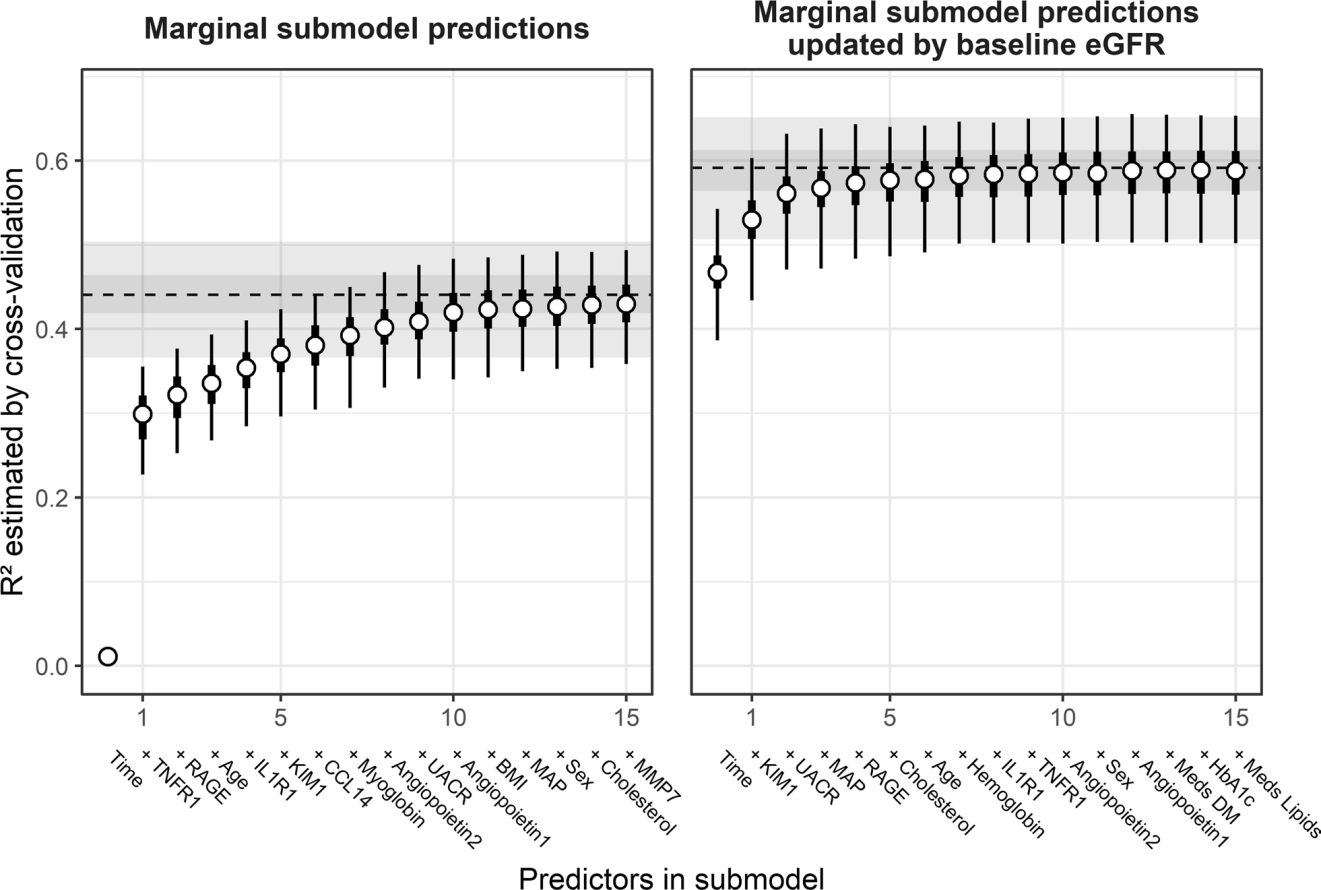
Approximation of main model by incremental submodels using the top 15 predictors, defined according to the ranking of variables by increase in cross-validated RMSE. The dashed line (posterior median $R^{2}$) and the dark and light grey shaded areas (50% and 95% BCI) indicate the full model performance in terms of cross-validated $R^{2}$. For submodels, the points indicate the posterior median $R^{2}$, thick and thin bars give 50% and 95% BCIs, respectively. The left panel depicts results when baseline eGFR is used as part of the longitudinal outcome vector, the right panel results when baseline eGFR is used to update predictions for post-baseline eGFR. The variables used in the submodels increase from left to right, starting with Intercept and time, then adding the first predictor according to the ranking (TNFR1 and KIM1, respectively), then adding the next predictor (RAGE and UACR, respectively), and so on. In particular, in the right panel the results show the added predictive performance for the predictors on top of baseline eGFR. The ordering shown is the ordering obtained across all cross-validation folds

The variable ordering is reported in Additional file 1: Table S6 and the corresponding incremental submodel performances are shown in Fig. 4 for the cross-validated $R^{2}$ (Additional file 1: Figure S6 shows cross-validated RMSE). The results corroborated the important roles of TNFR1, RAGE and age for objective 1, while KIM1 and UACR ranked as the most relevant predictors for objective 2 when incorporating baseline eGFR for predictions. This reflected their different roles for the prediction of eGFR trajectories: markers like TNFR1 and RAGE were relevant as a replacement of baseline eGFR and predictive of values close in time to baseline, while KIM1 and UACR were predictive of the future eGFR decline. Only few predictors were sufficient to approximate the performance of the full main model for both objectives, while the remaining predictors did not improve prediction performance substantially and were largely exchangeable. This is particularly evident for the prediction of future eGFR decline, in which case only KIM1 and to a lesser extent UACR provided substantial added predictive value on top of baseline eGFR.

## Discussion

In this study, we used Bayesian linear mixed modeling in a cohort of people with DM and moderately reduced eGFR to validate and discern the ability of a set of established serum protein biomarkers to predict eGFR trajectories. We found that in particular TNFR1 and RAGE contributed to the estimation of baseline eGFR values, while KIM1 and the clinical marker UACR were predictive for the future eGFR decline. This is in line with the current understanding of these markers. TNFR1 constitutes a marker of filtration, RAGE of general inflammatory response. On the other hand, KIM1 reflects kidney damage and thus plays an important role in the prediction of eGFR decline. Protein biomarkers slightly improved predictive performance in addition to clinical predictors alone. Nevertheless, only few predictors were sufficient to achieve similar performance to the full set of predictors. Baseline eGFR had a strong impact on predictive performance on top of all other variables. Studies like ours, bringing together a strong set of potential predictors for eGFR and evaluating their performance in a large cohort, are important to narrow down research efforts. Future work focused on improving our understanding of the most relevant protein biomarkers and their individual contributions to the prediction of eGFR decline may help to make them more clinically relevant in the treatment of CKD in people with DM.

The results from this work corroborate conclusions from our earlier studies that many biomarkers were associated with baseline eGFR, but that this association with eGFR diminished with increasing follow-up time [10, 15]. This indicated that the clinical utility of the biomarkers remained low compared to eGFR. A possible exception would be KIM1, which consistently demonstrated added value for the prediction of eGFR trajectories on top of baseline eGFR across a wide population at different CKD stages. Furthermore, as TNFR1 showed the strongest association with baseline eGFR it may be relevant to refine the accuracy with which the current disease status of an individual can be determined. Having multiple outcome related variables as opposed to a single measurement increases the reliability of an individual’s disease diagnosis and reduces issues with replicability of the results.

The findings from our work are in line with other studies. The investigations by Niewczas et al. and Gerstein et al. were used to define the pool of candidate biomarkers for our study [12, 13, 16]. While these studies also used selection techniques to identify markers important for predictions, they focused on the predictive abilities of the markers. On the other hand, our study tried to disambiguate the roles of the markers found in those studies in the prediction of longitudinal eGFR trajectories, which reflect their systemic biological functions.

The KidneyIntelX model was recently derived and validated as a prognostic tool for eGFR decline based on electronic health records, clinical predictors such as eGFR and the plasma biomarkers TNFR1, TNFR2 and KIM1 [17]. The investigators evaluated the predictions for a composite outcome of eGFR decline of 5 ml/min/1.73m^2^ per year or more, 40% or more sustained decline, or kidney failure within five years in biobanked plasma samples from two cohorts. We identified similar biomarkers in this study and were able to discern how they affect predictions by using a longitudinal outcome rather than a classification outcome.

Other investigators evaluated the KidneyIntelX risk score for the prediction of therapy response on longitudinal eGFR trajectories in a multinational cohort of people with diabetic kidney disease [14]. Treatment with the SGLT-2 inhibitor was found to reduce the KidneyIntelX score over time, and changes in the score from baseline to one year were associated with disease progression. The baseline status of an individual was important as people with higher baseline scores experienced more events compared to those with lower baseline scores. Therefore, an accurate diagnosis of the current disease state is relevant to predicting future disease progression. Our work similarly corroborates the importance of baseline eGFR for predictions of future eGFR decline.

Recent investigations of data from the multinational CANVAS study, a randomized trial assessing the effect of the SGLT2-inhibitor Canagliflozin on cardiovascular and kidney outcomes, also focused on TNFR1, TNFR2 and KIM1 as potential biomarkers [35, 36]. The studies found associations of TNFR1 and TNFR2 with progression of albuminuria, but did not show an association of KIM1 with albuminuria. Furthermore, Canagliflozin led to a modest attenuation of serum levels of TNFR1 and a decrease of KIM1 levels over time, indicating potential as markers for treatment response. The evidence from these studies complements our work, in which we found TNFR1 and KIM1 to be most promising candidates for eGFR prediction from a broad set of established biomarkers.

Other studies established ${\beta }_{2}$-microglobulin as another potentially interesting filtration marker for prediction of rapid renal function decline [19, 37]. While we did not measure this marker for our analysis, it was also shown to be highly correlated to TNFR1 (another marker of filtration) in these studies, which may serve as replacement in our analysis.

Our study has some limitations. The analysis cohort comprised people with mixed types of DM, but we can assume that most had type 2 DM. Our cohort baseline was the first in-person follow-up of the GCKD cohort rather than the actual enrolment visit due to sample availability. This potentially introduced bias due to people being lost to follow-up between the GCKD enrolment and our baseline. The death rate was low and the demographics of our study cohort showed largely similar characteristics as expected from the actual GCKD inclusion criteria. For these reasons, we assume that the loss-to-follow-up is largely not associated with study outcomes, and that the impact on our analysis results is low. Due to the limited sample availability, there were fewer follow-ups per person available to our analysis. We attempted to mitigate associated problems of large intra-individual variability by the use of mixed models for longitudinal eGFR values as outcome, rather than modeling surrogate endpoints. The GCKD cohort is a national study with participants from Germany, therefore representing a Caucasian population. However, since our results are in accordance and extend several other studies, we believe the findings to be generalizable to a broader population, or at least may foster further research in other settings.

Strengths of our study include the almost complete follow-up of the GCKD cohort and the low amount of missing data. The serum biomarkers in our study were pre-selected via available prior evidence, thus representing a strong set of predictors for eGFR decline. The Bayesian analysis used shrinkage priors to identify important predictors, while incorporating uncertainty about missing data and model fit. Furthermore, by updating the predictions by baseline eGFR we were able to discern for which parts of the longitudinal trajectory the variables were predictive, without being unduly influenced by the presence of baseline eGFR as independent variable.

In conclusion, we found that different serum protein biomarkers serve different roles for the prognostication of eGFR trajectories. These results may help to focus research efforts for such markers to improve understanding of their functions in the pathophysiology of CKD in people with DM and to make them more relevant to clinical applications.

## Supplementary Information

**Additional file 1: Table S1.** Protein biomarker measurement availability for analysis. **Table S2.** Protein biomarker measurement issues. **Table S3.** Convergence of Bayesian mixed modelsused in our study. **Table S4.** Cross-validated model performance by follow-up. **Table S5.** Coefficient posteriorsof main model. **Table S6.** Variable rankingsestimated via cross-validation according to contribution to prediction of eGFR values. **Figure S1.** Power analysisvia simulation. **Figure S2.** Measured protein biomarker concentrationsused for analysis(log2 transformed). **Figure S3.** Spearman correlationsbetween variablesin the analysis(based in pairwise complete observations). **Figure S4.** Residualsfor main BLMM. **Figure S5.** Coefficient posteriorsfor main model using Horseshoe shrinkage priorsand clinical and protein biomarkersaspredictors. **Figure S6.** Approximation of main model by incremental submodelsusing the top 15 predictors, defined according to the ranking of variablesby increase in cross-validated RMSE.

## Data Availability

The data that support the findings of this study are not openly available due to restrictions on availability, and inquiries about data usage can be sent to the corresponding author.
