# Supplementary material for: Different roles of protein biomarkers predicting eGFR trajectories in people with chronic kidney disease and diabetes mellitus: a nationwide retrospective cohort study
Source: Cardiovasc Diabetol. 2023 Mar 29;22:74. doi: 10.1186/s12933-023-01808-5 (PMC10061741; doi:10.1186/s12933-023-01808-5)
Supplement: Supplementary file 1 — Additional file 1: Table S1. Protein biomarker measurement availability for analysis. Table S2. Protein biomarker measurement issues. Table S3. Convergence of Bayesian mixed modelsused in our study. Table S4. Cross-validated model performance by follow-up. Table S5. Coefficient posteriorsof main model. Table S6. Variable rankingsestimated via cross-validation according to contribution to prediction of eGFR values. Figure S1. Power analysisvia simulation. Figure S2. Measured protein biomarker concentrationsused for analysis(log2 transformed). Figure S3. Spearman correlationsbetween variablesin the analysis(based in pairwise complete observations). Figure S4. Residualsfor main BLMM. Figure S5. Coefficient posteriorsfor main model using Horseshoe shrinkage priorsand clinical and protein biomarkersaspredictors. Figure S6. Approximation of main model by incremental submodelsusing the top 15 predictors, defined according to the ranking of variablesby increase in cross-validated RMSE. [file 12933_2023_1808_MOESM1_ESM.pdf]

Supplementary Material

Different roles of protein biomarkers predicting eGFR trajectories in people with chronic kidney disease and diabetes mellitus: a nationwide retrospective cohort study

Michael Kammer, PhD[1,2], Andreas Heinzel, MS[1], Karin Hu, MS[1], Heike Meiselbach, PhD[3], Mariella Gregorich, MS[1,2], Martin Busch, MD[4], Kevin L. Duffin, PhD[5], Maria F. Gomez, PhD[6], Kai-Uwe Eckardt, MD[3,7], Rainer Oberbauer, PhD[1] for the BEAt-DKD consortium

[1] Department of Internal Medicine III, Division of Nephrology and Dialysis, Medical University of Vienna, Vienna, Austria

[2] Center for Medical Data Science, Institute of Clinical Biometrics, Medical University of Vienna, Vienna, Austria

[3] Department of Nephrology and Hypertension, Friedrich-Alexander Universität Erlangen-Nürnberg, Erlangen, Germany

[4] Department of Internal Medicine III, University Hospital Jena, Friedrich-Schiller Universität, Jena, Germany

[5] Lilly Research Laboratories, Eli Lilly and Company, Indianapolis, IN, USA

[6] Lund University Diabetes Centre, Department of Clinical Sciences, Lund University, Malmö, Sweden

[7] Department of Nephrology and Medical Intensive Care, Charité Universitätsmedizin Berlin, Berlin, Germany

Corresponding author

Rainer Oberbauer, MD, PhD

Medical University of Vienna, Währinger Gürtel 18-20, A-1090 Vienna, Austria

T: +43 1 4040043900

rainer.oberbauer@meduniwien.ac.at

Table of contents

Table of contents 2

1. Extended statistical methods 3

Multiple imputation 3

Bayesian linear mixed model (BLMM) implementation 3

Model update by baseline eGFR 4

Submodel projections 4

Cross validation 5

Sensitivity analyses 5

2. Supplementary Tables 7

3. Supplementary Figures 15

4. References 22

1. Extended statistical methods

This section provides further details on the statistical analyses.

Multiple imputation

We used the multiple imputations by chained equations method to generate multiply imputed datasets. To this end, we extended the modeling dataset by information on why specific protein measurements were missing (i.e. missing due to a high coefficient of variation or technical issues). The imputation model then comprised all clinical and biomarker variables of interest, auxiliary variables to ensure missingness at random and increase accuracy of the imputations, and the outcome (eGFR at baseline and estimated eGFR slope from a frequentist linear mixed model) to ensure congeniality of imputation and analysis models (1). As imputation method we employed predictive mean matching with distance aided donor selection (2). We assessed convergence of the imputation model by graphically inspecting traceplots of the Markov chains, which showed satisfactory mixing of the Markov chains. Twenty imputed datasets were sampled from the model, since the overall amount of missingness was moderate. We chose to use multiple imputation prior to modeling to deal with missing data to provide a common base for all models evaluated in this study, rather than incorporating imputation into the Bayesian modeling procedure. The latter would have used different variable sets and likely led to incongruous imputations between the different sets of models, conflating the goals of the study (i.e. identification of predictors).

Bayesian linear mixed model (BLMM) implementation

We used Student-t distributed priors for the univariable and clinical models, with 3 degrees of freedom, mean zero and a scale parameter of 5. The regularized horseshoe priors in the main model used a Student-t distributed global shrinkage parameter with 2 degrees of freedom and scale set to $\frac{0.1}{\sqrt{n}}$, where 0.1 represents a prior assumption on the ratio of non-zero coefficients in the model, and n is the number of coefficients (3). The local shrinkage parameter used a Student-t distribution with 1 degree of freedom and scale 1. For all variance (half Student-t with 3 degrees of freedom and minimal scale 2.5, with final scaling depending on the scale of the random effect variable) and correlation parameters (Lewandowski-Kurowicka-Joe distribution with eta set to 1, i.e. all correlation matrices are equally likely a priori) the default, weakly informative priors as implemented

in the brms package were used. The results were not sensitive regarding the hyperparameters for priors in sensitivity analyses (results not shown), but the final values were chosen to optimize model convergence (i.e. minimize divergent transitions in the sampling algorithm).

We fitted all models on the 20 imputed datasets using three Markov chains for sampling using the No-U-Turn-Sampler (NUTS) algorithm implemented in the STAN software. The univariable and clinical model used 3000 samples (1000 for warmup) per chain, with a thinning rate of 2 to save memory. The main model using horseshoe priors required more samples to provide adequate convergence diagnostics, and used 4000 samples (1000 for warmup) per chain, with a thinning rate of 5. The adapt_delta parameter to control the target average proposal acceptance probability in NUTS was set to 0.95. Higher values removed all divergent transitions, but slowed down sampling too much to be feasible for the main analysis (also see Supplementary Table S 3).

Model update by baseline eGFR

We updated posterior predictions of a BLMM by computing the best linear unbiased predictors of the random effects conditional on the observed baseline values for eGFR. In detail, given a draw $\widehat{y_0}$ from the (marginal) posterior predictive distribution of eGFR at baseline of a new individual, and corresponding draws from the posteriors of the residual variance σ_ϵ^2, the random intercept variance σ_0^2 and the random effects covariance σ_{01}^2, the expected random effects were updated as

$$b_0 = \frac{\sigma_0^2(y_0 - \widehat{y_0})}{\sigma_0^2 + \sigma_\epsilon^2} \text{ and } b_1 = \frac{\sigma_{01} b_0}{\sigma_0^2},$$

where b_0 and b_1 denote the updated random intercept and slope. The initial predictions $\widehat{y_t}$ for later time points t were then updated by $\widehat{y_t}' = \widehat{y_t} + b_0 + t b_1$. Similar methodology was recently outlined in (4).

Submodel projections

Model projections for BLMM followed the methods outlined in (5) and implemented in the projpred R package. Our workflow was as follows: in each iteration of the cross-validation procedure, we re-fitted the full (reference) model on the training data and obtained a variable ranking determined by the drop in performance when projecting onto

a model excluding each variable in turn. This was done for the full model without (objective 1) and with updating by baseline eGFR (objective 2) separately, both of which yield different variable rankings due to the impact of baseline eGFR on the predictions. Then, incremental subsets of variables according to this ranking were used to define the submodel posteriors via projection. First, we projected the full model onto a submodel comprising only an intercept term and time. Then, the first predictors according to the ranking were added (main term and interaction term) to the submodel. Again, the first predictor differed for the objective 1 and objective 2. The remaining predictors were added according to the ranking one by one to obtain a sequence of nested submodels. To evaluate the performance of the projected submodels we used the unseen holdout data from the cross-validation, which we also used to update the submodel predictions via baseline eGFR. To speed up computations we did not use all posterior samples available but only a subset of 500. Sensitivity analysis indicated that higher sample numbers did not yield different results, but reduced variability.

Cross validation

We used repeated cross-validation to minimize the variance of the performance estimates, which we found to be highly variable in a single 5-fold cross-validation run. The cross-validation was on a per-person basis, i.e. all observations of a person from all imputed datasets were assigned to a single cross-validation fold.

Sensitivity analyses

We conducted sensitivity analyses on subsamples of all imputed datasets to asses different hyperparameters for the model priors and different modeling assumptions (results not shown for brevity). Overall, there was little impact of different parameters, likely due to the relatively large sample size and moderate number of predictors. Changing scale parameters to be wider, or degrees of freedom to 1 to 3 for the Student-t distributions used in the models did not markedly affect the results. Similarly, different parameters for the regularized horseshoe prior led to unchanged results. Specifically the expected amount of non-zero coefficients, which controls sparsity, was changed from 0.1 to 0.5 with no discernible impact on the coefficient posteriors. Changing the degrees of freedom between 1 and 3 for the Student-t distributions of the global and local shrinkage parameters did not affect the coefficient posteriors either, but resulted in

varying numbers of divergent transitions.

Using weakly informative priors instead of the shrinkage Horseshoe prior did not improve prediction performance of the main BLMM, but led to more diffuse coefficient posteriors. A model using Student-t distributed errors to deal with the heavy tails of the residual distribution led to largely similar posteriors but required more computational resources to fit.

2. Supplementary Tables

Supplementary Table S 1: Protein biomarker measurement availability for analysis. In total, measurements for samples from 838 people were conducted. The following table gives the number of persons who were analyzed (those missing due to measurement issues were multiply imputed, see also Supplementary Table S 2), or whose samples were truncated for analysis. Percentages relate to the total number of participants.

Biomarker	Analysed*	Truncated for analysis†
a1Microglobulin	825 (98%)	4 (<1%)
Angiopoietin1	838 (100%)	0 (0%)
Angiopoietin2	763 (91%)	0 (0%)
CCL11	750 (89%)	302 (36%)
CCL14	811 (97%)	0 (0%)
CCL15	770 (92%)	57 (7%)
CCL5	838 (100%)	0 (0%)
Chemerin	824 (98%)	3 (<1%)
Fas	823 (98%)	0 (0%)
FasLigand	815 (97%)	105 (13%)
Galectin3	838 (100%)	0 (0%)
GDF15	723 (86%)	2 (<1%)
IL1R1	822 (98%)	0 (0%)
KIM1	838 (100%)	30 (4%)
MMP7	825 (98%)	19 (2%)
Myoglobin	838 (100%)	0 (0%)
RAGE	823 (98%)	0 (0%)
TNFR1	822 (98%)	0 (0%)
uPA	820 (98%)	0 (0%)

* Number of people for which available measurements had acceptable coefficient of variation between duplicate measurements (≤15%), and no technical measurement issues.

† Number of people for which the analysed data was truncated at the limits of the standard ranges.

Supplementary Table S 2: Protein biomarker measurement issues. The following table comprises information on all biomarker measurements (may be multiple per person) and issues during measurement. Percentages refer to the corresponding total number of measurements per marker. Number of measurements differ between the different measurement platforms (KIM1 Elisa 979, Luminex 5-plex 960 and Luminex 13-plex 1026).

**Biomarker**	**Measurements**	**Issues**		
		**%CV***	**Range†**	**Other‡**
a1Microglobulin	1026	19 (2%)	9 (1%)	4 (<1%)
Angiopoietin1	960	8 (1%)	0 (0%)	0 (0%)
Angiopoietin2	1026	30 (3%)	0 (0%)	72 (7%)
CCL11	1026	126 (12%)	450 (44%)	16 (2%)
CCL14	960	43 (5%)	0 (0%)	0 (0%)
CCL15	1026	127 (12%)	79 (8%)	3 (<1%)
CCL5	960	2 (<1%)	0 (0%)	0 (0%)
Chemerin	1026	37 (4%)	3 (<1%)	3 (<1%)
Fas	1026	23 (2%)	0 (0%)	2 (<1%)
FasLigand	1026	61 (6%)	143 (14%)	3 (<1%)
Galectin3	960	3 (<1%)	0 (0%)	0 (0%)
GDF15	1026	196 (19%)	2 (<1%)	3 (<1%)
IL1R1	1026	29 (3%)	0 (0%)	2 (<1%)
KIM1	979	20 (2%)	36 (4%)	0 (0%)
MMP7	1026	36 (4%)	29 (3%)	2 (<1%)
Myoglobin	960	1 (<1%)	0 (0%)	0 (0%)
RAGE	1026	26 (3%)	0 (0%)	2 (<1%)
TNFR1	1026	26 (3%)	0 (0%)	3 (<1%)
uPA	1026	62 (6%)	2 (<1%)	3 (<1%)

* Coefficient of variation > 15% between duplicate measurements on same measurement plate. Excluded / imputed for analysis.

† Measurement outside standard concentration range. Truncated for analysis.

‡ Technical measurement issues (too low bead count, other). Excluded / imputed for analysis.

Supplementary Table S 3: Convergence of Bayesian mixed models used in our study. Overall, the convergence of our models was satisfactory. The $\hat{R}$ statistic measures convergence by comparing the between- and within-Markov chain estimates of model parameters. Values should be close to 1 – much higher values (i.e. > 1.01) indicate bad mixing of the chains and that a model has not converged. We report here the median and 97.5th percentile of the $\hat{R}$ statistic across all model parameters, treating multiple imputations as additional chains.

Divergent transitions are reported by the Hamiltonian MCMC sampler implemented in Stan. These may occur with highly varying posterior curvature, preventing the sampler from fully exploring the posterior and thereby potentially biasing the results. Ideally, there are no divergent transitions. The numbers of divergent transitions in all our models was low (at most 5), and could be eliminated by increasing the adaption process of the step size during sampling (adapt_delta parameter > 0.95). However, due to the increasing computational demand this was not implemented in the full analysis using cross-validation and multiple imputation, but only investigated in a single imputation. Results did not differ, so we chose to report findings with a low number of divergences using adapt_delta 0.95 as a compromise.

**Model**	$\hat{R}$		**Divergent transitions (%)**
	**Median**	**97.5th quantile**	
All univariable models	1.001	1.007	<0.001%
Clinical reference	1.000	1.004	0%
Main	0.999	1.003	<0.001%
Main model refitted in all cross-validation iterations	0.999	1.005	<0.001%

Supplementary Table S 4: Cross-validated model performance by follow-up. Results are reported as median posterior values for cross-validated R^2 and root mean squared error (RMSE), along with a 95% BCI in parentheses. The values were computed using the observed eGFR value at a given follow-up and the model's marginal predictions using only fixed effects. Note that the baseline performances after update by baseline eGFR are reported only for completeness and are to be considered over-optimistic. They were not included in the reported post-baseline performances in the main manuscript.

**Model**	**Follow-up**	**Performance**		**Performance after update by baseline eGFR**	
		**R^2**	**RMSE**	**R^2**	**RMSE**
**Clinical**	Baseline	0.08 [0.05, 0.13]	10.74 [9.87, 11.45]	0.88 [0.82, 0.92]	2.95 [2.38, 3.62]
	Year 2	0.17 [0.11, 0.23]	10.69 [9.98, 11.86]	0.61 [0.52, 0.68]	7.61 [6.43, 9.18]
	Year 4	0.21 [0.14, 0.28]	13.79 [12.31, 15.20]	0.53 [0.42, 0.61]	10.28 [9.11, 11.51]
**Main (Combined)**	Baseline	0.44 [0.35, 0.50]	8.12 [7.40, 8.88]	0.81 [0.72, 0.88]	3.99 [3.21, 4.90]
	Year 2	0.46 [0.39, 0.53]	8.96 [8.12, 10.18]	0.63 [0.55, 0.68]	7.54 [6.44, 8.59]
	Year 4	0.43 [0.33, 0.52]	11.35 [9.64, 12.80]	0.57 [0.48, 0.66]	9.82 [8.58, 11.29]

Supplementary Table S 5: Coefficient posteriors of main model. Results are reported as median and 95% Bayesian credible interval (BCI) for the main term of each variable (baseline coefficient), and its interaction with observation time (slope coefficient). Coefficients are given for standardized variables, i.e. correspond to a change of one standard deviation of the variable.

Variable	Baseline coefficient (95% BCI)	Slope coefficient (95% BCI)
Intercept	43.15 [41.88, 44.34]	-0.26 [-0.94, 0.13]
Age	-1.35 [-1.90, -0.79]	-0.07 [-0.27, 0.06]
Sex (Female)	-1.19 [-2.65, 0.05]	-0.09 [-0.59, 0.15]
BMI	0.86 [0.16, 1.48]	0.00 [-0.15, 0.16]
Smoking (Ever)	0.10 [-0.17, 0.69]	0.03 [-0.09, 0.23]
MAP	0.13 [-0.14, 0.75]	-0.38 [-0.59, -0.17]
Cholesterol	-0.09 [-0.70, 0.18]	0.28 [0.06, 0.50]
HbA_{1c}	-0.03 [-0.49, 0.23]	-0.07 [-0.26, 0.06]
Hemoglobin	0.05 [-0.23, 0.61]	0.27 [0.04, 0.48]
UACR*	0.17 [-0.14, 0.87]	-0.70 [-0.92, -0.48]
Medication Bloodpressure (Yes)	0.01 [-0.72, 1.15]	-0.15 [-0.90, 0.20]
Medication Diabetes Mellitus (Yes)	-0.01 [-0.86, 0.54]	-0.28 [-0.86, 0.08]
Medication Lipid lowering (Yes)	-0.01 [-0.75, 0.45]	0.06 [-0.15, 0.47]
a1Microglobulin*	-0.15 [-0.83, 0.14]	-0.06 [-0.28, 0.07]
Angiopoietin1*	0.85 [0.13, 1.47]	0.06 [-0.08, 0.29]
Angiopoietin2*	0.94 [0.25, 1.54]	0.05 [-0.08, 0.27]
CCL11*	0.01 [-0.31, 0.39]	0.00 [-0.14, 0.15]
CCL14*	-1.59 [-2.24, -0.90]	0.06 [-0.08, 0.30]
CCL15*	-0.46 [-1.13, 0.04]	0.00 [-0.15, 0.17]
CCL5*	0.02 [-0.27, 0.51]	0.03 [-0.10, 0.22]
Chemerin*	-0.27 [-0.92, 0.08]	-0.03 [-0.21, 0.10]
Fas*	0.47 [-0.08, 1.35]	0.00 [-0.17, 0.20]
FasLigand*	-0.11 [-0.71, 0.15]	0.03 [-0.09, 0.23]

Galectin3*	-0.00 [-0.41, 0.34]	0.03 [-0.10, 0.22]
GDF15*	-0.01 [-0.48, 0.33]	0.05 [-0.09, 0.29]
IL1R1*	1.47 [0.78, 2.13]	0.03 [-0.11, 0.25]
KIM1*	0.02 [-0.30, 0.61]	-0.79 [-1.04, -0.55]
MMP7*	-0.45 [-1.21, 0.05]	-0.01 [-0.21, 0.16]
Myoglobin*	-1.68 [-2.33, -1.02]	0.18 [-0.02, 0.41]
RAGE*	-1.77 [-2.46, -1.05]	-0.20 [-0.45, 0.02]
TNFR1*	-4.41 [-5.32, -3.51]	0.06 [-0.11, 0.38]
uPA*	0.04 [-0.23, 0.53]	0.05 [-0.07, 0.26]

* variable was log2-transformed during modeling

Supplementary Table S 6: Variable rankings estimated via cross-validation according to contribution to prediction of eGFR values. In each cross-validation iteration variables were ranked by the increase in the root-mean-squared-error of the holdout data. This table provides summaries of the ranks across all cross-validation folds. The final ranking was defined by the median of ranks across all cross-validation folds.

Variable	Median of ranks	Mean of ranks	Best rank	Worst rank
**Ranked via marginal predictions**				
TNFR1	1	1	1	1
RAGE	2	2.04	2	3
Age	3	3.84	3	6
IL1R1	4	4.4	2	7
KIM1	5	5.24	3	10
CCL14	6	6.64	3	11
Myoglobin	7	6.84	3	11
Angiopoietin2	8	7.96	6	11
UACR	9	8.44	5	12
Angiopoietin1	10	9.92	6	15
BMI	11	11.36	7	17
MAP	12	12.52	9	18
Sex	13	13.72	7	23
Cholesterol	16	17.28	12	29
MMP7	16	15.8	12	24
CCL15	17	18.72	12	30
Fas	17	18.64	12	29
Chemerin	18	18.64	14	26
Hemoglobin	18	17.56	11	28
Meds DM	19	19.24	14	30
a1Microglobulin	20	20.04	12	27
HbA_{1c}	22	23.56	16	31
uPA	24	24.04	17	30
Smoking Ever	25	24.08	14	31
FasLigand	25	25.24	20	31
Meds BP	25	24.76	16	31
Meds Lipids	26	25.76	19	30
CCL5	27	25.92	20	31
GDF15	27	26.52	19	31
Galectin3	29	27.6	20	31
CCL11	30	28.68	21	31

Ranked via marginal post-baseline predictions updated by baseline eGFR				
KIM1	1	1.32	1	2
UACR	2	1.68	1	2
MAP	4	3.76	3	6
RAGE	4	3.84	3	7
Cholesterol	5	5.16	3	9
Age	6	6.64	4	10
Hemoglobin	7	7.48	4	12
IL1R1	8	8.12	5	13
TNFR1	10	12.44	6	30
Angiopoietin2	11	13.28	8	27
Sex	11	10.72	6	17
Angiopoietin1	13	13.64	6	30
Meds DM	13	13.6	9	26
HbA_{1c}	15	16.56	10	27
Meds Lipids	18	18.04	10	28
BMI	20	21.28	12	30
Chemerin	20	20.52	11	31
GDF15	20	20.56	11	31
Meds BP	20	20.32	10	28
a1Microglobulin	21	21.28	13	31
FasLigand	21	21.2	12	28
MMP7	21	20	11	31
Smoking Ever	22	21.36	12	30
Myoglobin	23	22.4	11	31
uPA	23	22.56	14	31
Fas	24	23.52	15	30
CCL14	25	23.44	11	31
Galectin3	25	23.36	11	31
CCL11	26	24.88	11	30
CCL5	27	25.64	16	31
CCL15	29	27.4	17	31

3. Supplementary Figures

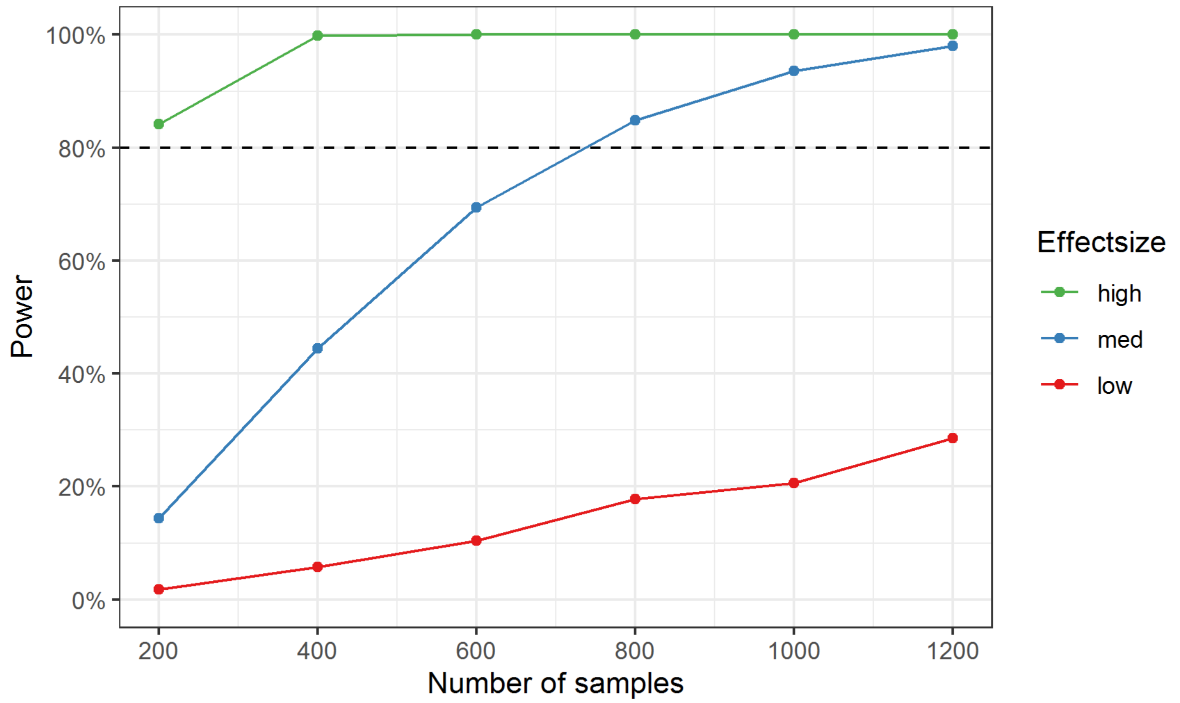

Supplementary Figure S 1: Power analysis via simulation. Effect sizes low / medium / high correspond to standardized regression coefficients of 0.25 / 0.5 / 1 and increases in R^2 due to the biomarker by <1% / 1% / 5%. The power curves were derived from 500 simulation runs.

Considerations on sample size were based on published data from the PROVALID cohort (6). We assumed that the analysis consists of a pool of 20 competing biomarkers, which are added separately to a panel of 10 clinical covariates in order to predict the eGFR slope analysed by a linear regression model. At various sample sizes, we assessed the power to detect a single biomarker. We used a conservative Bonferroni correction and divided the nominal two-sided significance level of 0.05 by the number of candidate biomarkers (leading to a two-sided significance level of 0.0025). We assumed that biomarkers are normally distributed across patients (based on PROVALID data, this is plausible after log2 transformation). The effect sizes were defined in terms of a standardized regression coefficient, i.e. the change in the eGFR slope corresponding to a change of one standard deviation of the biomarker. Based on PROVALID data, we

defined strong effects to be comparable to the effect of KIM1 (standardized absolute coefficient of 1) in our preceding analysis, while medium and low effect sizes were defined as standardized absolute coefficients of 0.5 and 0.25, respectively. Clinical covariates were modelled as normal distributed with a correlation between the predictors of 0.1, in accordance with PROVALID data. We specified the correlation of the biomarker and the clinical covariates in terms of average correlation to the clinical predictors as 0.1, in accordance with PROVALID data. In PROVALID the average decline of eGFR was around -2 mL/min/1.73m²/year with a standard deviation of around 4. The explained variation of a model predicting the eGFR slope based on clinical covariates only was moderate (around 35%). We assumed a similar standard deviation of the slope (4) and an average effect size of the clinical covariates to achieve an R^2 of the clinical model of 30%.
Note that this power analysis primarily focuses on the effect of a biomarker on eGFR trajectories via the eGFR slope, since effects on the current (baseline) eGFR levels are much easier to identify. For effect sizes of the biomarker comparable to our earlier studies (6), 800 observations would yield around 85% power for our analysis.

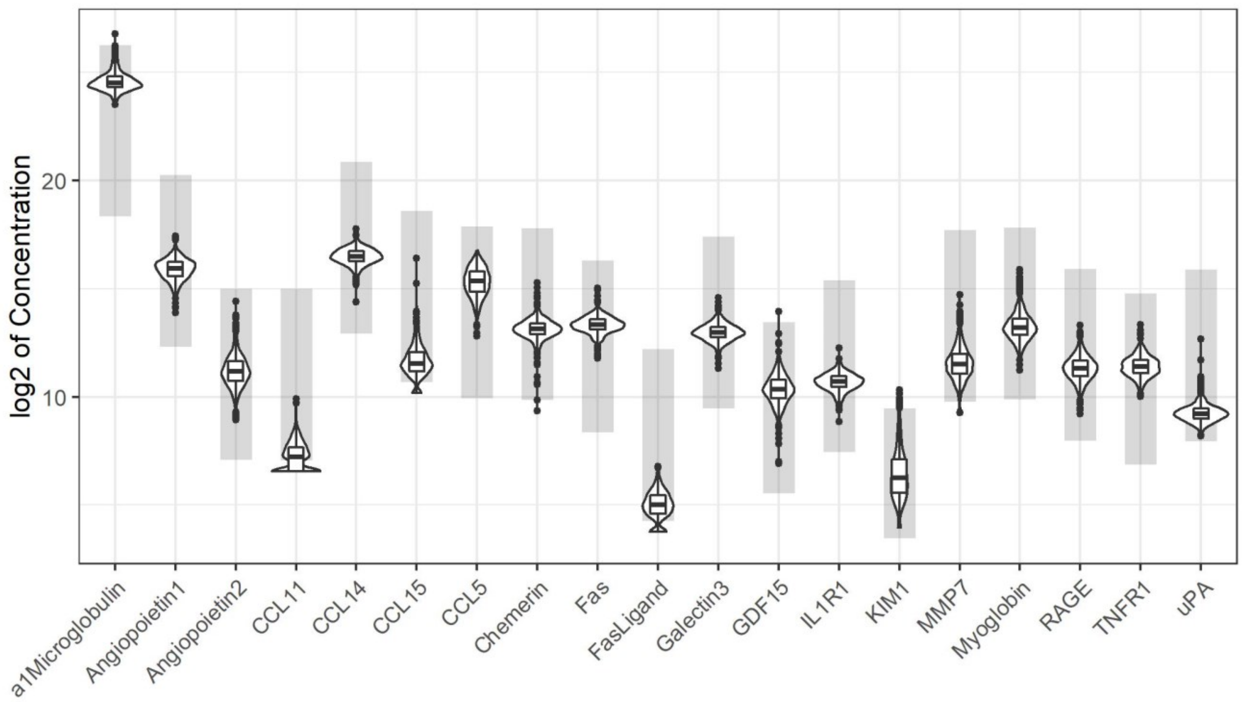

Supplementary Figure S 2: Measured protein biomarker concentrations used for analysis (log2 transformed). Data are shown as boxplots, the distributions are depicted as underlying violinplots (i.e. density plots mirrored around the y-axes of the boxplot). The grey bars indicate the measurement standard range for each biomarker.

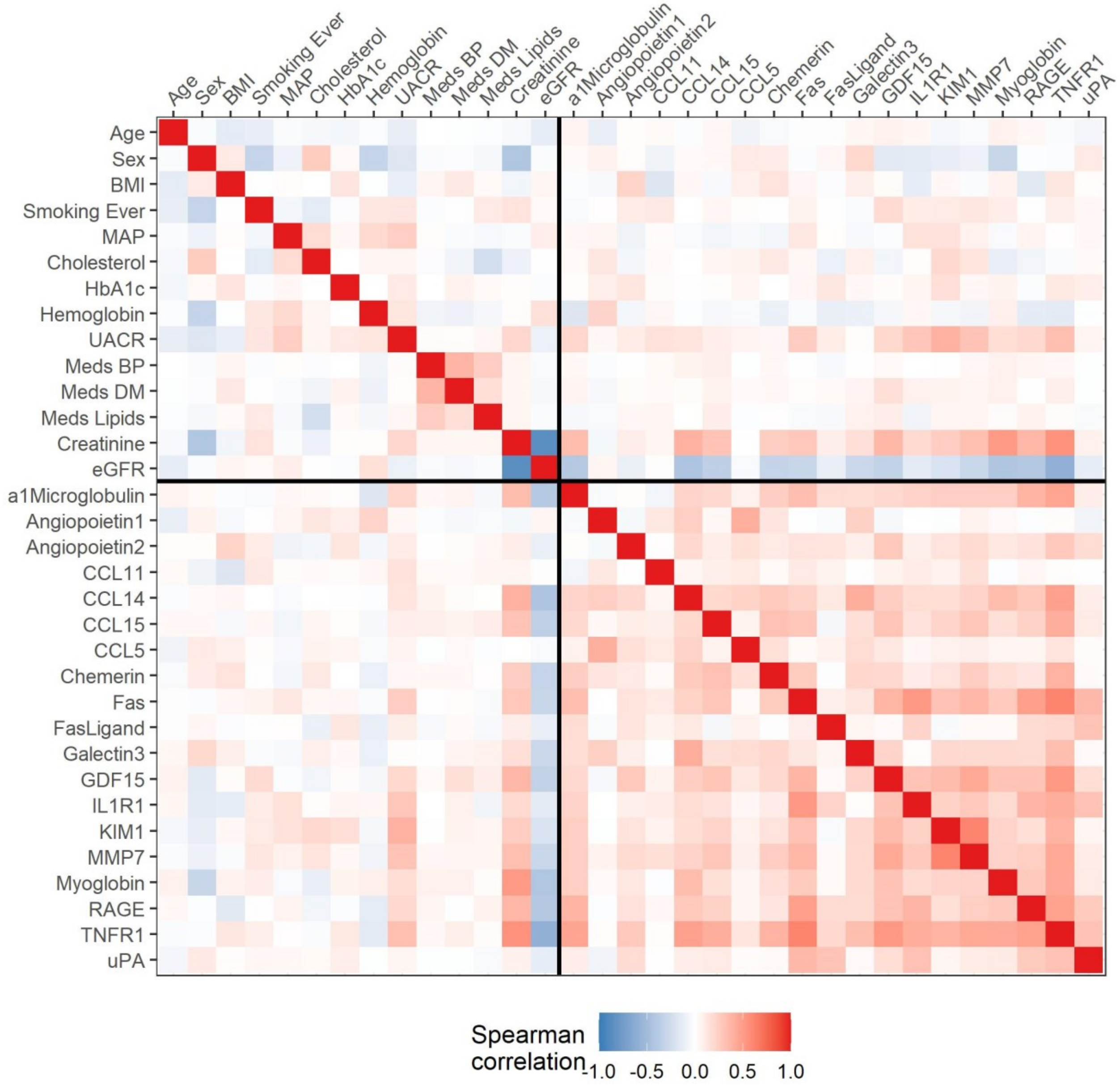

Supplementary Figure S 3: Spearman correlations between variables in the analysis (based in pairwise complete observations). The black lines separate clinical variables and protein biomarkers.

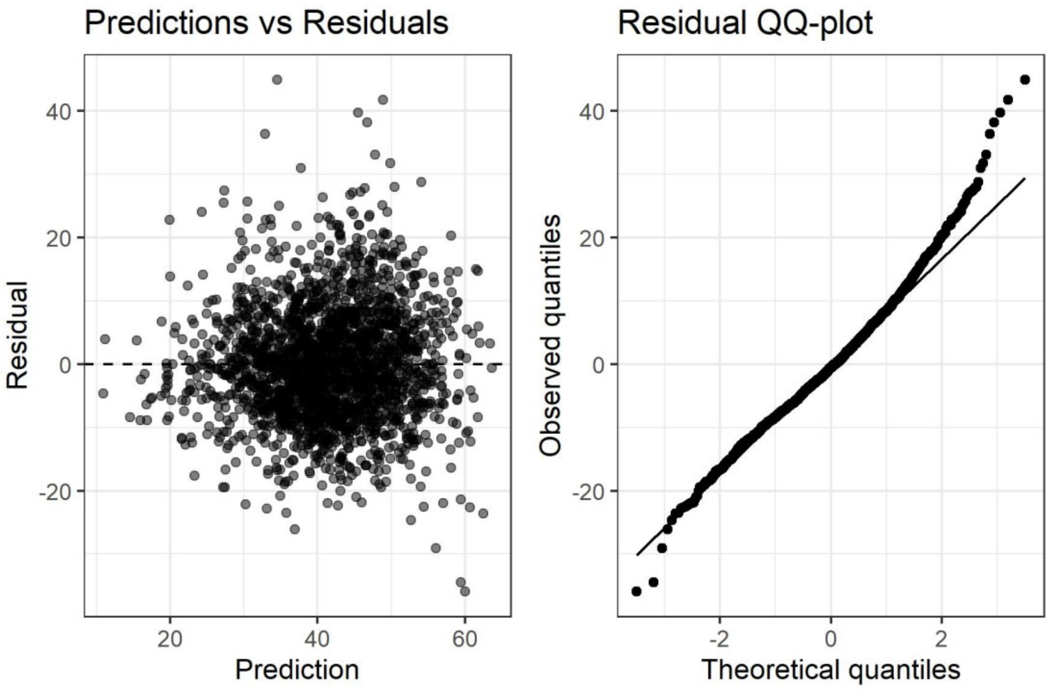

Supplementary Figure S 4: Residuals for main BLMM. Left panel shows marginal median posterior predictions vs marginal median posterior residual for each person and timepoint. Right panel shows a quantile-quantile plot of the residuals.

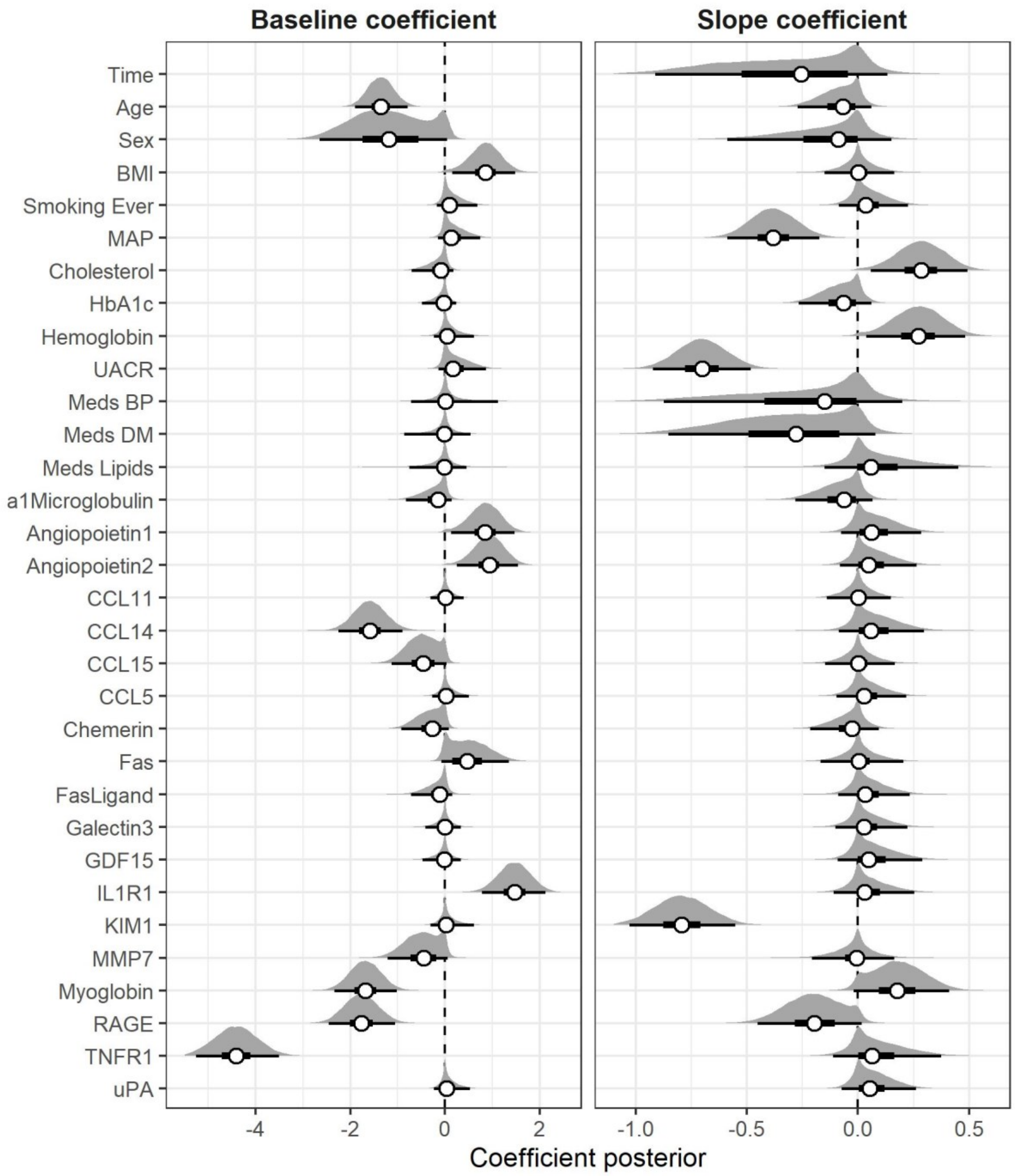

Supplementary Figure S 5: Coefficient posteriors for main model using Horseshoe shrinkage priors and clinical and protein biomarkers as predictors. Data were standardized, such that coefficients correspond to a change of one standard deviation. Intercept not shown, all protein markers and UACR were log2-transformed. Note the different x-axis scale in both panels. For exact numerical results, see Supplementary Table S 5.

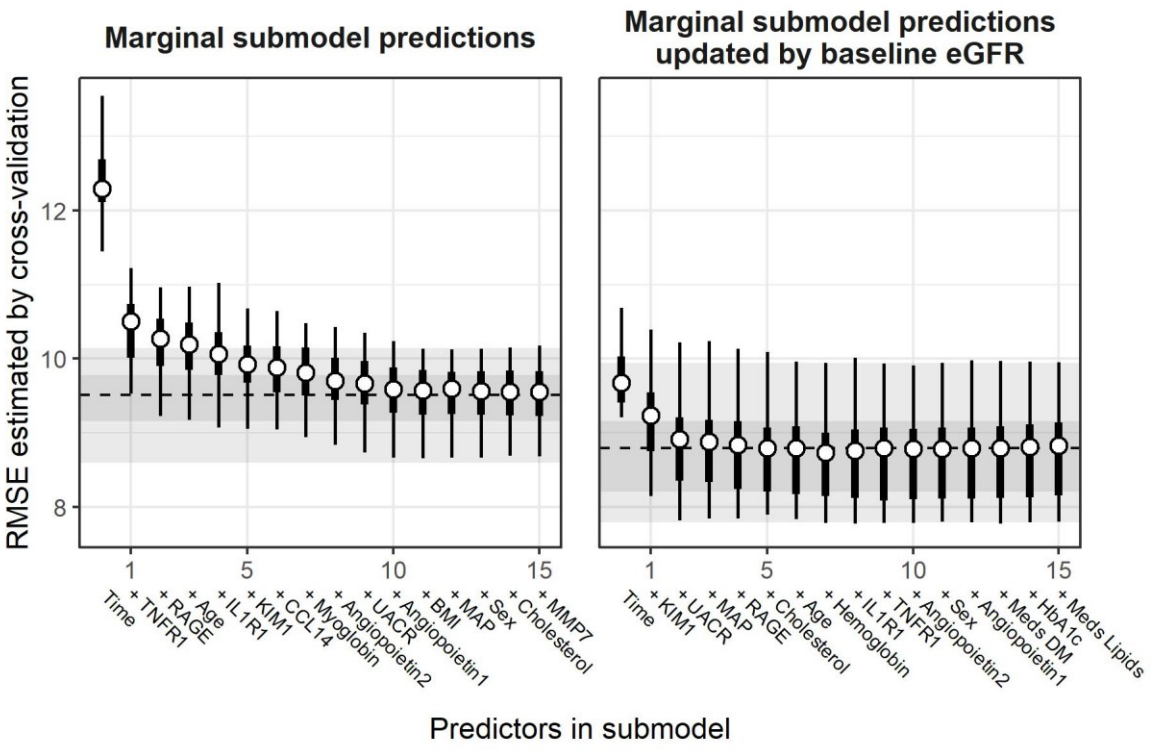

Supplementary Figure S 6: Approximation of main model by incremental submodels using the top 15 predictors, defined according to the ranking of variables by increase in cross-validated RMSE. The dashed line (posterior median RMSE) and the dark and light grey shaded areas (50% and 95% BCI) indicate the full model performance. For submodels, the points indicate the posterior median RMSE, thick and thin bars give 50% and 95% BCIs, respectively. The left panel depicts results when baseline eGFR is used as part of the outcome, the right panel results when baseline eGFR is used to update predictions for post-baseline eGFR. The variables used in the submodels increase from left to right, starting with Intercept and time, then adding the first predictor (TNFR1 and KIM1, respectively), then adding the next predictor (RAGE and UACR, respectively), and so on.

4. References

1. Hayati Rezvan P, Lee KJ, Simpson JA. The rise of multiple imputation: a review of the reporting and implementation of the method in medical research. BMC Med Res Methodol. 2015;15:30.
2. Siddique J, Belin TR. Multiple imputation using an iterative hot-deck with distance-based donor selection. Stat Med. 2008;27(1):83-102.
3. Piironen J, Vehtari A. Sparsity information and regularization in the horseshoe and other shrinkage priors. Electronic Journal of Statistics. 2017;11(2):5018-51.
4. Gregorich M, Heinzel A, Kammer M, Meiselbach H, Böger C, Eckardt K-U, et al. A prediction model for the decline in renal function in people with type 2 diabetes mellitus: study protocol. Diagn Progn Res. 2021;5(1):19.
5. Catalina A, Bürkner P-C, Vehtari A. Projection predictive inference for generalized linear and additive multilevel models. arXiv preprint arXiv:201006994. 2020.
6. Heinzel A, Kammer M, Mayer G, Reindl-Schwaighofer R, Hu K, Perco P, et al. Validation of Plasma Biomarker Candidates for the Prediction of eGFR Decline in Patients With Type 2 Diabetes. Diabetes Care. 2018;41(9):1947-54.
